# Advanced EEG signal classification for neural prosthetic devices using metaheuristic and deep learning techniques

**DOI:** 10.3389/fdgth.2025.1706660

**Published:** 2026-01-06

**Authors:** Thippagudisa Kishore Babu, Damodar Reddy Edla, Suresh Dara, Mohan Allam

**Affiliations:** 1 Department of Computer Science and Engineering, National Institute of Technology, Cuncolim, Goa, India; 2 School of Computer Science and Engineering, VIT-AP University, Amaravati, Andhra Pradesh, India

**Keywords:** coati optimization algorithm (COA), deep learning, EEG signal classification, feature selection, motor imagery, neural prosthetic devices

## Abstract

**Introduction:**

For neural prosthetic devices, accurate classification of high dimensional electroencephalography (EEG) signals is significantly impaired by the existence of redundant and irrelevant features that deteriorate the classifier generalization and computation efficiency. This work presents a new and unified optimal-driven framework to challenge these issues and improve EEG-based MI signal decoding.

**Methods:**

The proposed method combines a modified feature selection model of coati optimization algorithm (COA) and different machine/deep learning classifiers. The novelty of the COA is its dynamic and parameter-free adaptation mechanism, in association with opposition-based learning a better exploration exploitation balance can be maintained in high-dimensional feature space. The generated optimized feature subsets are then employed to train a battery of classifiers such as support vector machines (SVM), random forests (RF), convolutional neural networks (CNN) and recurrent neural networks (RNN) for motor imagery task recognition. In experiments, we verify SSRC on commonly used benchmark EEG datasets such as the PhysioNet Motor Movement/Imagery dataset.

**Results:**

The experimental results showed that the COA + CNN model had the best performance of classification. The model demonstrated a classification accuracy of 96.8% of prediction, with precision at moderate AH hour and predicted as either being more likely to discharge or remain in care = 96.4%, recall = 96.9% and F1-score = 96.6%. This presents a remarkable 6.5% gain in classification accuracy over the best rival feature selection technique and significantly outperformed conventional metaheuristic algorithms such as PSO (90.3% accuracy) and GA (89.7% accuracy) as well as filter-type techniques such as mRMR (86.8%) and ReliefF (84.3%).

**Discussion/Conclusion:**

The combined evolved metaheatistic for feature subset selection with deep learning architectures is a powerful approach for an accurate classification EEG signals. The findings confirm that the COA-based approach provides a robust, computationally-efficient, and scalable method for achieving high-accuracy classification—essential for promoting the reliability and real-time operation of future neural prosthetic control systems.

## 1 Introduction

The convergence of artificial intelligence (AI), biomedical signal processing, and assistive technologies has created transformative opportunities in the development of intelligent healthcare systems [1]. Among the most impactful innovations in this domain are neural prosthetic devices, which enable individuals with motor disabilities to control external devices, such as robotic arms or wheelchairs, through the interpretation of their brain signals. This technological advancement holds tremendous promise for improving the quality of life of individuals affected by spinal cord injuries, stroke-induced paralysis, and neuromuscular disorders. Central to the operation of neural prosthetics is the accurate decoding of brain activity into machine-interpretable commands. Electroencephalography (EEG) has emerged as a preferred modality for neural prosthetic control due to its non-invasive nature, high temporal resolution, cost-effectiveness, and ability to capture real-time brain dynamics. The EEG-based brain–computer interfaces (BCIs), particularly those relying on motor imagery (MI) tasks, are widely used in prosthetic control, where users imagine limb movements to generate distinctive neural patterns that can be classified into commands for prosthetic actuation [2]. Despite these advancements, the successful deployment of such systems faces significant challenges. The EEG signals are often outrageously non-stationary, noisy, and participant-dependent, resulting in low signal-to-noise ratios. In addition, real-world EEG datasets typically contain a high number of irrelevant and redundant features, which not only increase computational resources but also degrade the performance of classification algorithms. The inclusion of unnecessary features leads to the curse of dimensionality, resulting in increased model complexity, slower inference times, and reduced generalization to unseen data [3].

To overcome these limitations, feature selection has become an indispensable step in EEG signal analysis and broader medical data classification tasks. The objective is to identify and retain the most informative subset of features that contribute to class separation while discarding irrelevant or noisy attributes. However, the performance of traditional feature selection methods is often hindered by their reliance on static heuristics or fixed parameter settings. Moreover, the dynamic and evolving nature of biomedical signals, especially EEG, requires adaptive and flexible optimization strategies that can balance global search and local refinement in high-dimensional feature spaces. In this context, the development of adaptive, metaheuristic-based feature selection techniques, particularly those with dynamic search behavior, is of growing interest. These methods not only reduce dimensionality and computational cost but also significantly enhance the performance of classifiers used in medical diagnosis and neural control systems. The coati optimization algorithm (COA) [4], a recent bioinspired metaheuristic, has shown promising results in various global optimization tasks. However, its standard implementation lacks dynamic adaptability and remains sensitive to initial parameter settings—issues that are critical when applied to complex, noisy datasets like EEG.

To address these challenges, this paper proposes a COA that introduces opposition-based learning and parameter-free adaptation to enhance both global and local search capabilities. When integrated with robust machine learning and deep learning classifiers, this hybrid framework aims to provide a scalable and accurate solution for real-time EEG signal classification and other biomedical diagnostic tasks.

### 1.1 Problem statement and research gap

Despite significant progress in neural prosthetic systems and EEG-based classification, several critical gaps persist, listed as follows: (i) high-dimensionality and feature redundancy in EEG and medical datasets reduce classifier performance and slow down processing time, making real-time deployment challenging; (ii) static optimization techniques lack adaptability during the feature selection process and often require extensive parameter tuning, limiting their scalability across datasets with varying structures and noise profiles; (iii) existing metaheuristic algorithms show potential but fall short in dynamic medical environments, where real-time adaptability and minimal latency are essential for usability; and, finally, (iv) integration of dynamic feature selection with deep learning models in EEG-driven systems presents a barrier to achieving high classification accuracy with low computational overhead [5].

To overcome these limitations, this paper presents a hybrid framework that combines COA for feature selection, which incorporates dynamic opposition-based learning strategies. This improves exploration and exploitation without requiring any manual fine-tuning of parameters. Then, a comprehensive classification stage is employed that uses both traditional classifiers, such as support vector machines (SVMs) and random forests (RFs), and deep learning models, such as convolutional neural networks (CNNs) and recurrent neural networks (RNNs), to decode EEG signals corresponding to imagined motor tasks. Next, multiple real-world EEG medical datasets are evaluated to validate performance in diverse dimensional spaces.

The framework aims to reduce dimensionality while improving classification accuracy, computational efficiency, and suitability for real-time prosthetic control.

### 1.2 Key contributions

This study introduces a unified optimization-driven framework for EEG-based neural prosthetic control, with the following key contributions:

A novel COA-based approach is introduced for feature selection. Unlike existing algorithms, such as particle swarm optimization (PSO), genetic algorithm (GA), ant colony optimization (ACO), and minimum redundancy maximum relevance (mRMR), the proposed COA incorporates dynamic candidate generation and parameter-free adaptation, enabling a stronger balance between exploration and exploitation in high-dimensional EEG feature spaces.
An integrated hybrid classification pipeline combining COA with deep learning models (CNN and RNN) and conventional models (SVM, RF) is developed for improved motor imagery EEG classification.
A 6.5% improvement in classification accuracy over the best competing method across benchmark EEG datasets is achieved, emphasizing the effectiveness of dynamic feature selection.
A scalable and real-time-oriented feature selection and classification framework is proposed for neural prosthetic applications, supporting rapid interpretation of user intention.

The remainder of this paper is structured as follows: Section 2 presents a detailed review of existing evolutionary, swarm-based, and filter-based feature selection methods for EEG analysis. Section 3 describes linear programming formulation for proposed evolutionary method. In the Section 4, the dynamic coati optimization algorithm and its integration into the EEG classification framework. Section 5, describing the proposed method with EEG data pre-processing, feature extraction, feature selection using COA, classification with deep learning with illustrative examples, and dataset descriptions. Section 6 outlines the experimental setup, and parameter configurations, performance analysis, comparisons with existing methods. Section 7 concludes the work and outlines future research directions.

## 2 Related study

Numerous studies have explored feature selection and classification techniques for EEG signal analysis, particularly in the context of BCIs and neural prosthetic systems. These approaches are classified into evolutionary methods and filter-based methods.

### 2.1 Filter-based methods

In contrast, filter-based methods, such as mRMR [6] and ReliefF [7], offer computationally efficient alternatives by ranking features based on statistical measures. mRMR seeks to maximize relevance to class labels while minimizing redundancy among the selected features. Although effective in many applications, its reliance on linear assumption about feature interactions restricts its performance in capturing non-linear EEG dynamics. Similarly, ReliefF estimates feature importance by measuring differences between neighboring instances but struggles with high-dimensional and noisy datasets, reducing its robustness for real-time EEG classification tasks. The chi-square test [8] evaluates the dependence between features and class labels using the chi-square statistics. Although fast, it assumes independence between features and often underperforms in high-dimensional biomedical datasets. The F-score [9] measures the discriminative power of individual features by comparing inter-class and intra-class variances. It is widely used but overlooks feature interactions, thereby limiting its effectiveness in complex EEG signals.

The field of EEG-based motor imagery classification has seen substantial development in feature extraction, dimensionality reduction, and classifier optimization. Traditional filter-based feature selection approaches, such as mRMR, ReliefF, F-score ranking, and Fisher discriminant ratio, are commonly used to identify discriminative EEG features. Although these methods are computationally efficient, they are limited by their inability to capture non-linear interactions that occur among EEG channels.

### 2.2 Evolutionary methods

To overcome these limitations, evolutionary and swarm-based algorithms [6, 10–13] have been extensively explored for biomedical feature selection.

Evolutionary methods, such as GAs and PSO, have gained attention for their capability to handle complex, high-dimensional feature spaces. GAs [13], based on biological evolution principles of selection, crossover, and mutation, have been used to select optimal EEG features for motor imagery classification; however, they often suffer from premature convergence and high computational overhead, especially in noisy and non-stationary EEG environments. Similarly, PSO [12], inspired by the social behavior of bird flocking, has been employed to search for optimal solutions. Although widely used in EEG feature selection, PSO may converge prematurely and fail to reach global optima. ACO [11] has shown promising results in feature selection tasks but suffers from long convergence times and sensitivity to parameters. Simulated annealing (SA) probabilistically explores the solution space and allows the acceptance of worse solutions at early stages to escape local optima. Despite being more robust, it is typically slower than population-based algorithms [10]. However, it can be sensitive to parameter tuning and may get trapped in local optima, limiting its generalization in dynamic EEG patterns.

However, most existing algorithms lack dynamic exploration ability and rely heavily on fixed parameters, reducing their effectiveness when applied to complex neural datasets with high variability. In contrast, the proposed COA introduces parameter-free adaptation and dynamic candidate generation inspired by coati behaviors. This significantly enhances the search process and provides a more robust feature selection mechanism for high-dimensional EEG classification.

## 3 Linear programming formulation

Despite significant progress in biomedical signal classification [14], particularly in EEG-based neural prosthetic systems, the efficient selection of informative features from high-dimensional datasets remains a critical challenge. The presence of irrelevant or redundant features not only reduces classification performance but also imposes significant computational and latency burdens. These factors hinder real-time decision-making, which is essential to assistive applications such as prosthetic control. To address this challenge, we formulate the feature selection task as a linear programming (LP) task that aimed at selecting an optimal subset of features that maximizes classification relevance under multiple real-world constraints. Before LP formulation, we define few important variables for a clear understanding, as follows:

d: Total number of available features;
$z_{j}\in \{0,1\}$: Binary variable indicating whether feature j is selected;
$s_{j}\in [0,1]$: Importance (or relevance) score of feature j;
$c_{j}$: Computational cost associated with feature j;
$r_{j}$: Latency contribution of feature j;
$g_{j}\in \{0,1\}$: Indicator if feature j belongs to a required group (e.g., spatial);
B: Total allowable computational budget;
R: Maximum allowable latency;
k: Maximum number of features to select; and
$G_{\min}$: Minimum number of features required from a specific group.

The objective function is to maximize the overall relevance of the selected features, which is shown in Equation 1.

$$\max_{z}\sum_{\;j=1}^{d}s_{j}z_{j}$$ (1)

Subject to the following constraints:

$$\sum_{\;j=1}^{d}c_{j}z_{j}\leq B$$ (2)

$$\sum_{\;j=1}^{d}r_{j}z_{j}\leq R$$ (3)

$$\sum_{\;j=1}^{d}z_{j}\leq k$$ (4)

$$\sum_{\;j=1}^{d}g_{j}z_{j}\geq G_{\min}$$ (5)

where constraint Equation 2 ensures that the total computational cost of selected features does not exceed the predefined budget B. Constraint Equation 3 restricts the total latency contribution of the selected features to remain within the real-time requirements of the system. Afterward, constraint Equation 4 limits the number of selected features to control model complexity and prevent overfitting. Finally, constraint Equation 5 enforces diversity by requiring a minimum number of features from an essential group.

## 4 Coati optimization algorithm

The COA is a population-based metaheuristic inspired by the intelligent foraging behavior of coatis. It is particularly effective for binary optimization problems, such as feature selection in biomedical signal processing (e.g., EEG classification). In this framework, each candidate solution is modeled as a coati, and their behavior in searching for optimal food sources is abstracted into a computational framework.

Each phase of the COA plays a distinct role in guiding the search process. Initially, the COA generates a diverse population of binary solutions. It then assesses solution quality using fitness evaluation. Next, it updates the position of a coati with respect to the leader in the territorial movement phase. Further, it explores previously unexplored regions by flipping solution values in the phase of opposition-based learning. Finally, it retains the best solution in the leader update phase.

### 4.1 Coati encoding and initialization

Each coati $X_{i}$ is represented as a binary vector of length d, where d is the number of features in the dataset. The binary vector determines whether each feature is selected (1) or not selected (0) for inclusion in the classification model, and it is mathematically represented in Equation 6.

$$X_{i}=[x_{i1},x_{i2},x_{i3},\ldots ,x_{id}],\;x_{ij}\in \{0,1\}$$ (6)

Initialization is performed by assigning each bit $x_{ij}$ randomly using a uniform probability, as depicted in Equation 7.

$$x_{ij}^{\left(0\right)}=\left\{ \begin{array}{cc} 1,\; & \text{if }rand<p\;\\ 0,\; & \text{otherwise}\; \end{array}\right.$$ (7)

Here, rand∼U(0,1) and p is typically set to 0.5 to encourage equal selection probability across features. This step ensures diversity in the initial population, which is crucial for effectively covering the entire search space.

### 4.2 Fitness evaluation and dominant male selection

After initializing the population, each coati’s performance is assessed using a fitness function mentioned in Equation 8.

$$f_{i}=f(X_{i})$$ (8)

The coati with the best fitness (e.g., lowest classification error) is selected as the dominant male, as mentioned in Equation 9.

$$X^{*}=\arg\min_{i}f(X_{i})$$ (9)

This leader guides the rest of the population in the subsequent steps of the optimization process.

### 4.3 Territorial movement

In this phase, each coati updates its position by probabilistically imitating the dominant male. The switching probability that controls whether a bit is adopted from the leader is given by Equation 10.

$$S_{ij}=r_{1}\cdot (1-r_{2}\cdot x_{ij}^{\left(t\right)}),\;r_{1},r_{2}∼U(0,1)$$ (10)

Based on this probability, each bit of a coati is updated using the rule given in Equation 11.

$$x_{ij}^{\left(t+1\right)}=\left\{ \begin{array}{cc} x_{ij}^{*},\; & \text{if }rand<S_{ij}\;\\ x_{ij}^{\left(t\right)},\; & \text{otherwise}\; \end{array}\right.$$ (11)

This strategy combines exploitation (learning from the leader) with exploration (retaining current traits), enabling a dynamic and balanced search.

### 4.4 Opposition-based learning

To improve search diversity and avoid local optima, each coati generates an opposite solution by flipping its bits, as represented in Equation 12.

$${\bar{x}}_{ij}=\left\{ \begin{array}{cc} 1,\; & \text{if }x_{ij}=0\;\\ 0,\; & \text{otherwise}\; \end{array}\right.$$ (12)

The better of the original and opposite solution is retained as shown in Equation 13.

$$X_{i}=\left\{ \begin{array}{cc} {\bar{X}}_{i},\; & \text{if }f({\bar{X}}_{i})<f(X_{i})\;\\ X_{i},\; & \text{otherwise}\; \end{array}\right.$$ (13)

This mechanism ensures that unexplored areas of the solution space are evaluated for potential improvement.

### 4.5 Leader update

After all coatis have been updated, the fitness values are re-evaluated, and a new dominant male is selected if a better solution has emerged. The mathematical representation of the leader update is provided in Equation 14.

$$X^{*}=\arg\min_{i}f(X_{i})$$ (14)

This step ensures that the global best solution is always preserved and used to guide the next iteration. Finally, the optimization continues until either a maximum number of iterations is attained or a minimal improvement over recent generations is achieved.

## 5 Proposed method

We introduce a novel EEG signal classification framework for neural prosthetic devices by employing the COA to enhance feature selection and improve classification accuracy. The objective is to translate motor intentions from EEG signals into control commands for assistive devices, which is a core requirement in neural prosthetics for individuals with motor impairments. The proposed method is divided into several key stages: EEG data acquisition and preprocessing, feature extraction, feature selection using COA, classification using deep learning models, and performance evaluation.

### 5.1 EEG data acquisition and preprocessing

The system begins with the acquisition of EEG signals from participants performing motor imagery tasks such as imagining hand or foot movement. These tasks are selected to replicate control signals for prosthetic device movement. EEG signals are acquired using a multi-channel headset with high temporal resolution. Preprocessing steps such as bandpass filtering, artifact removal like eye blinks and muscle noise, and normalization are applied to enhance signal quality and reliability. Only clean and task-relevant segments are used for further analysis.

EEG signals were recorded while participants performed predefined motor imagery tasks, simulating limb movements. The preprocessing phase involved noise filtering, artifact removal, and normalization to enhance signal quality. Let the EEG signal acquired from C channels over T time samples be represented as a multivariate matrix as shown in Equation 15.

$$E=\left[\begin{array}{cccc} e_{11} & e_{12} & \cdots & e_{1T}\\ e_{21} & e_{22} & \cdots & e_{2T}\\ \vdots & \vdots & \ddots & \vdots \\ e_{C1} & e_{C2} & \cdots & e_{CT} \end{array}\right]$$ (15)

where $e_{ct}$ denotes the signal amplitude from channel c at time t. Preprocessing involves bandpass filtering, artifact removal, and z-score normalization as Equation 16.

$$e_{ct}^{\prime }=\frac{e_{ct}-\mu_{c}}{\sigma_{c}},\;\mu_{c}=\text{mean}(e_{c}),\;\sigma_{c}=\text{std}(e_{c})$$ (16)

### 5.2 Feature extraction

To convert raw EEG signals into informative patterns, we apply a combination of time-domain, frequency-domain, and spatial-domain feature extraction techniques. Features such as signal variance, power spectral density (PSD), common spatial patterns (CSP), and wavelet coefficients are extracted from each EEG channel. The resulting feature vectors are typically high-dimensional, which necessitates an effective selection mechanism to reduce redundancy and improve classification performance. The theoretical foundations of the proposed feature selection and extraction method with fitness function and updating strategies are summarized in Equations 17–28. For simplicity of reference, the meaning of each variable and abbreviation is given embedded in its respective equation.

Each EEG segment is transformed into a feature vector using domain-specific techniques, including time-domain, frequency-domain, and wavelet-domain analyses. The resulting feature vector for the ith trial is denoted by

$$F_{i}=[f_{1},f_{2},\ldots ,f_{d}]\in R^{d}$$ (17)

where d is the total number of extracted features. Given the high dimensionality of $F_{i}$, an optimal subset must be selected to improve model generalization and reduce redundancy.

### 5.3 Feature selection using COA

Given the high dimensionality of EEG feature vectors, feature selection is crucial for identifying the most relevant features for accurate classification. We employ the COA, a population-based binary metaheuristic inspired by the adaptive foraging behavior of coatis. Each solution (i.e., coati) is encoded as a binary string, where each bit indicates the inclusion or exclusion of a specific feature.

Initially, a population of coatis is randomly generated. The fitness of each coati is evaluated based on classification accuracy using a lightweight classifier on a validation subset. The coati with the highest accuracy is designated as the dominant male and guides the rest of the population. The coatis probabilistically adopt features from the leader using a switching probability mechanism that balances exploration and exploitation. To escape local optima, opposition-based learning is introduced, where opposite solutions are generated and accepted if they provide better performance. The Algorithm 1 iteratively updates the population and the leader until convergence or a maximum number of iterations is reached. Each coati $X_{i}$ is encoded as a binary string representing feature inclusion:

$$X_{i}=[x_{i1},x_{i2},\ldots ,x_{id}],\;x_{ij}\in \{0,1\}$$ (18)

where $x_{ij}=1$ implies selection of the jth feature. The fitness of each solution is computed using classification accuracy:

$$f(X_{i})=1-\text{Acc}(X_{i})$$ (19)

The best solution (dominant male) is defined as

$$X^{*}=\arg\min_{i}f(X_{i})$$ (20)

Each coati updates its bits based on a switching probability: ?>

$$S_{ij}=r_{1}\cdot (1-r_{2}\cdot x_{ij})$$ (21)

$$\begin{array}{rl} x_{ij}^{\left(t+1\right)} & =\left\{ \begin{array}{cc} x_{ij}^{*},\; & \text{if }rand<S_{ij}\;\\ x_{ij}^{\left(t\right)},\; & \text{otherwise}\; \end{array}\right. \end{array}$$ (22)

To enhance diversity, opposition-based learning is applied: ?>

$${\bar{x}}_{ij}=1-x_{ij}$$ (23)

$$\begin{array}{rl} X_{i}^{\left(t+1\right)} & =\left\{ \begin{array}{cc} {\bar{X}}_{i},\; & \text{if }f({\bar{X}}_{i})<f(X_{i})\;\\ X_{i},\; & \text{otherwise}\; \end{array}\right. \end{array}$$ (24)

After updating all individuals, the leader is updated:

$$X^{*}=\arg\min_{i}f(X_{i}^{\left(t+1\right)})$$ (25)

The process terminates when it reaches a maximum number of iterations. This optimization reduces the feature space significantly, retaining only those features that maximize classification performance while minimizing redundancy.

### 5.4 Classification with deep learning

The reduced feature set obtained from COA is then fed into a deep learning classifier—typically a CNN or an RNN, depending on the temporal characteristics of the data. CNNs are employed for spatial pattern learning across channels, while RNNs, such as LSTM units, are effective for learning temporal dependencies in EEG data. These models are trained on the selected features and evaluated using standard classification metrics. The optimized feature vector $F_{i}^{\prime }$ is constructed by selecting features where $x_{ij}=1$:

$$F_{i}^{\prime }=[f_{j}|x_{ij}=1]$$ (26)

A deep learning classifier C, such as a CNN or LSTM, is then trained:

$${\hat{y}}_{i}=C(F_{i}^{\prime };\Theta )$$ (27)

where ${\hat{y}}_{i}$ is the predicted class label and Θ represents the model parameters. The network is trained using cross-entropy loss:

$$L=-\sum_{i=1}^{N}y_{i}\log({\hat{y}}_{i})$$ (28)

Algorithm 1 **EEG signal classification using COA.**

**Require**: Raw EEG signal data, number of coatis *N*, max iterations *T*

**Ensure**: Classified motor intention labels

 1: **Step 1: EEG acquisition**

  Collect EEG signals from participants performing motor imagery tasks

 2: **Step 2: Preprocessing**

  Apply bandpass filtering, remove noise and artifacts, and normalize signals

 3: **Step 3: Feature extraction**

  Extract time-domain, frequency-domain, and spatial features

  Construct feature matrix $F\in R^{n\times d}$

 4: **Step 4: Initialize coati optimization algorithm (COA)**

 5: **for** i=1 to *N*

 6: Randomly generate binary solution $X_{i}\in \{0,1{\}}^{d}$

 7: Compute fitness $f\;(X_{i})=1-{\mathrm{Accuracy}}_{LOOCV}(X_{i})$

 8: **end for**

 9: **Step 5: Optimization loop**

 10: **for** t=1 to *T* **do**

 11: Identify the dominant male coati *X*_*best*_ with the lowest *f*(*X*)

 12: **for** each coati $X_{j}\neq X_{best}$ **do**

 13:  Update *X*_*j*_ based on the dominant male and dynamic behavior

 14:  Generate opposition solution ${\bar{X}}_{j}$

 15:  Evaluate fitness $f\;(X_{j})$ and $f\;({\bar{X}}_{j})$

 16:  Retain solution with better fitness

 17: **end for**

 18: **end for**

 19: **Step 6: Feature subset selection**

  Select the final best solution $X_{best}$

  Subset *F* to $F^{*}$ using selected features

 20: **Step 7: Classifier training and evaluation**

  Train classifiers (SVM, RF, CNN, RNN) using $F^{*}$ and labels *Y*

  Evaluate using cross-validation (accuracy, precision, recall, F1)

 21: **Step 8: Output**

  Report the best classifier for neural prosthetic control

### 5.5 Illustrative example #1

To illustrate the proposed framework, a numerical example is demonstrated on a simplified EEG dataset comprising five trials with six extracted features per trial. The objective is to classify EEG signals using selected features optimized by the COA.

#### 5.5.1 EEG feature matrix and class labels

Let the feature matrix $F\in R^{5\times 6}$ and corresponding class labels Y∈{0,1} be

$$\begin{array}{cc} F & =\left[\begin{array}{cccccc} 0.85 & 0.47 & 0.90 & 0.11 & 0.63 & 0.40\\ 0.78 & 0.50 & 0.88 & 0.14 & 0.60 & 0.42\\ 0.25 & 0.33 & 0.27 & 0.92 & 0.55 & 0.49\\ 0.24 & 0.36 & 0.23 & 0.89 & 0.53 & 0.50\\ 0.30 & 0.29 & 0.32 & 0.94 & 0.57 & 0.52 \end{array}\right],\\ Y & =\left[\begin{array}{ccccc} 1 & 1 & 0 & 0 & 0 \end{array}\right] \end{array}$$

#### 5.5.2 Coati initialization and feature encoding

Assume that three coatis are initialized using binary encoding to represent different feature subsets as below:

$$\begin{array}{rl} X_{1} & =[1,0,1,0,1,0]\;\text{( select }f_{1},f_{3},f_{5})\\ X_{2} & =[0,1,1,1,0,1]\;\text{( select }f_{2},f_{3},f_{4},f_{6})\\ X_{3} & =[1,1,0,1,0,1]\;\text{( select }f_{1},f_{2},f_{4},f_{6}) \end{array}$$

#### 5.5.3 Feature subsetting

The feature matrix for $X_{1}$ (selected features: 1, 3, 5) is given as follows:

$$F_{1}^{\prime }=\left[\begin{array}{ccc} 0.85 & 0.90 & 0.63\\ 0.78 & 0.88 & 0.60\\ 0.25 & 0.27 & 0.55\\ 0.24 & 0.23 & 0.53\\ 0.30 & 0.32 & 0.57 \end{array}\right]$$

#### 5.5.4 Fitness evaluation using LOOCV and 1-NN

The fitness value is computed using leave-one-out cross-validation (LOOCV) with a 1-nearest-neighbor (1-NN) classifier. We perform test on sample 1, Test: [0.85, 0.90, 0.63], and training on samples 2–5. Now, we compute Euclidean distances to $S_{2}$: $\sqrt{(0.85-0.78{)}^{2}+(0.90-0.88{)}^{2}+(0.63-0.60{)}^{2}}\approx 0.078$.

Similarly, we compute Euclidean distances to $S_{3}\;:\;\approx 0.846$, $S_{4}\;:\;\approx 0.865$, and $S_{5}\;:\;\approx 0.819$. Further, the true labels for $S_{1},S_{2},S_{3},S_{4}$, and $S_{5}$ are 1, 1, 0, 0, and 0, respectively. Then, the fitness can be defined as follows:

$$f(X_{i})=1-\text{Accuracy}(X_{i})$$

For $X_{1}$: all five samples were correctly classified

$$\text{Accuracy}(X_{1})=\frac{5}{5}=1.0\;\Rightarrow \;f(X_{1})=0.0$$

Similarly, for $X_{2}$: there is only one misclassification, $\text{Accuracy}(X_{2})=4/5=0.8\Rightarrow f(X_{2})=0.2$, and for $X_{3}$, there are two misclassifications, $\text{Accuracy}(X_{3})=3/5=0.6\;\Rightarrow \;f(X_{3})=0.4$.

#### 5.5.5 Coati optimization phases

The best-performing coati, $X_{1}$, becomes the dominant male. Binary switching and opposition-based learning are applied to update other coatis:

$${\bar{X}}_{3}=[0,0,1,0,1,0]\Rightarrow \text{selected: }f_{3},f_{5}$$

Assuming re-evaluation yields improved accuracy: $\text{Accuracy}({\bar{X}}_{3})=$ $0.9\Rightarrow f({\bar{X}}_{3})=0.1$.

#### 5.5.6 Final feature selection

After multiple iterations, the optimal feature subset selected by the best coati $X_{1}$ is

$$X_{1}=[1,0,1,0,1,0]\Rightarrow \text{Selected features: }f_{1},f_{3},f_{5}$$

The final reduced matrix is

$$F^{*}=\left[\begin{array}{ccc} 0.85 & 0.90 & 0.63\\ 0.78 & 0.88 & 0.60\\ 0.25 & 0.27 & 0.55\\ 0.24 & 0.23 & 0.53\\ 0.30 & 0.32 & 0.57 \end{array}\right]$$

### 5.6 Computational complexity

The computational complexity of the proposed EEG classification framework is primarily driven by the COA-based feature selection. The preprocessing and feature extraction steps require $O(n\cdot d\log d+d^{3})$ time, where n is the number of samples and d is the number of features. During the COA phase, initializing N coatis and evaluating their fitness using LOOCV with a 1-NN classifier incurs a cost of $O(N\cdot n^{2}\cdot k)$, where k denotes the average number of selected features. The main optimization loop, executed for T iterations, dominates the complexity with $O(T\cdot N\cdot n^{2}\cdot k)$.

### 5.7 Dataset description

In this study, we utilized the publicly available PhysioNet EEG Motor Movement/Imagery Dataset [15], which is specifically designed for evaluating EEG-based BCI systems. This dataset contains EEG recordings from 109 healthy individuals, performing various motor tasks, including both actual and imagined movements of the left fist and right fist, both fists simultaneously, both feet, and various combinations thereof.

EEG signals were recorded using the BCI2000 system at a sampling frequency of 160 Hz with 64 electrodes positioned according to the international 10–10 system (16). Each participant performed multiple sessions that included baseline resting states, motor execution, and motor imagery tasks. The data are provided in European Data Format (EDF), and the recordings are annotated with task markers for precise segmentation.

For this research, segments corresponding to motor imagery tasks were extracted, preprocessed, and used to construct feature matrices. These segments were then used as input to the proposed feature selection and classification pipeline. This dataset provides a comprehensive and diverse set of EEG signals that are well-suited for evaluating both traditional machine learning and deep learning models for neural prosthetic control.

### 5.8 Performance evaluation

Model performance is evaluated using standard metrics: ?>

$$\text{Accuracy}=\frac{\text{TP}+\text{TN}}{\text{TP}+\text{TN}+\text{FP}+\text{FN}}$$ (29)

$$\text{Precision}=\frac{\text{TP}}{\text{TP}+\text{FP}}$$ (30)

$$\text{Recall}=\frac{\text{TP}}{\text{TP}+\text{FN}}$$ (31)

$$\text{F1-score}=\frac{2\cdot \text{Precision}\cdot \text{Recall}}{\text{Precision}+\text{Recall}}$$ (32)

$$\text{Reduction rate}=1-\frac{\text{\# selected features}}{d}$$ (33)

The proposed system is evaluated using multiple EEG datasets (e.g., BCI Competition datasets, PhysioNet motor imagery datasets, and so on). Cross-validation is employed to ensure robustness, and performance is assessed using accuracy, precision, recall, F1-score, and feature reduction rate. The respective equations for these metrics are presented in Equations 29–33. Comparisons are made with other feature selection methods, such as GA [13], PSO [12], and traditional filter-based techniques.

The proposed method integrates efficient feature selection via the coati optimization algorithm with robust deep learning-based EEG classification. This hybrid framework significantly improves the responsiveness and accuracy of neural prosthetic systems, providing a reliable control interface for individuals with motor disabilities. The ability to interpret these signals with precision forms the foundation for converting brain activity into control commands for external devices such as robotic limbs. The classification of EEG signals remains one of the most complex tasks due to the non-stationary and noisy nature of signals, as well as the variability introduced by different individuals and recording conditions. Enhancing the accuracy and responsiveness of neural prosthetic systems requires the use of advanced signal processing techniques and intelligent classifiers that can handle such complexities.

## 6 Results and analysis

To evaluate the effectiveness of the proposed framework, experiments were conducted using the PhysioNet EEG motor imagery dataset. The parameter settings for the proposed framework are given in Table 1.

**Table 1:** Parameter settings for the proposed framework.

Component	Parameter setting
COA optimization	
Population size (N)	20
Number of iterations (T)	50
Encoding scheme	Binary (0/1 for feature selection)
Fitness evaluation	LOOCV with 1-NN
Opposition learning	Dynamic, based on dominant solution
Preprocessing	
Normalization method	Min–max normalization
Feature extraction	
Time-domain features	Mean, variance, skewness
Frequency-domain features	PSD, FFT
Spatial filtering	CSP
Classification models	
SVM kernel	RBF
Random forest	100 trees, Gini criterion
CNN	2 convolution layers, ReLU, MaxPooling
RNN (LSTM)	1 LSTM layer, 64 units, dropout = 0.3
Training details	
Batch size	32
Epochs	100
Optimizer	Adam
Learning rate	0.001

The model performance was assessed using metrics such as accuracy, precision, recall, and F1-score, averaged across multiple cross-validation folds. All experiments were implemented in Python using Scikit-learn, TensorFlow, and PyTorch libraries and executed on a system equipped with an Intel i7 processor, 16 GB RAM, and an NVIDIA RTX GPU.

### 6.1 Proposed method performance analysis

**Figure 1:**
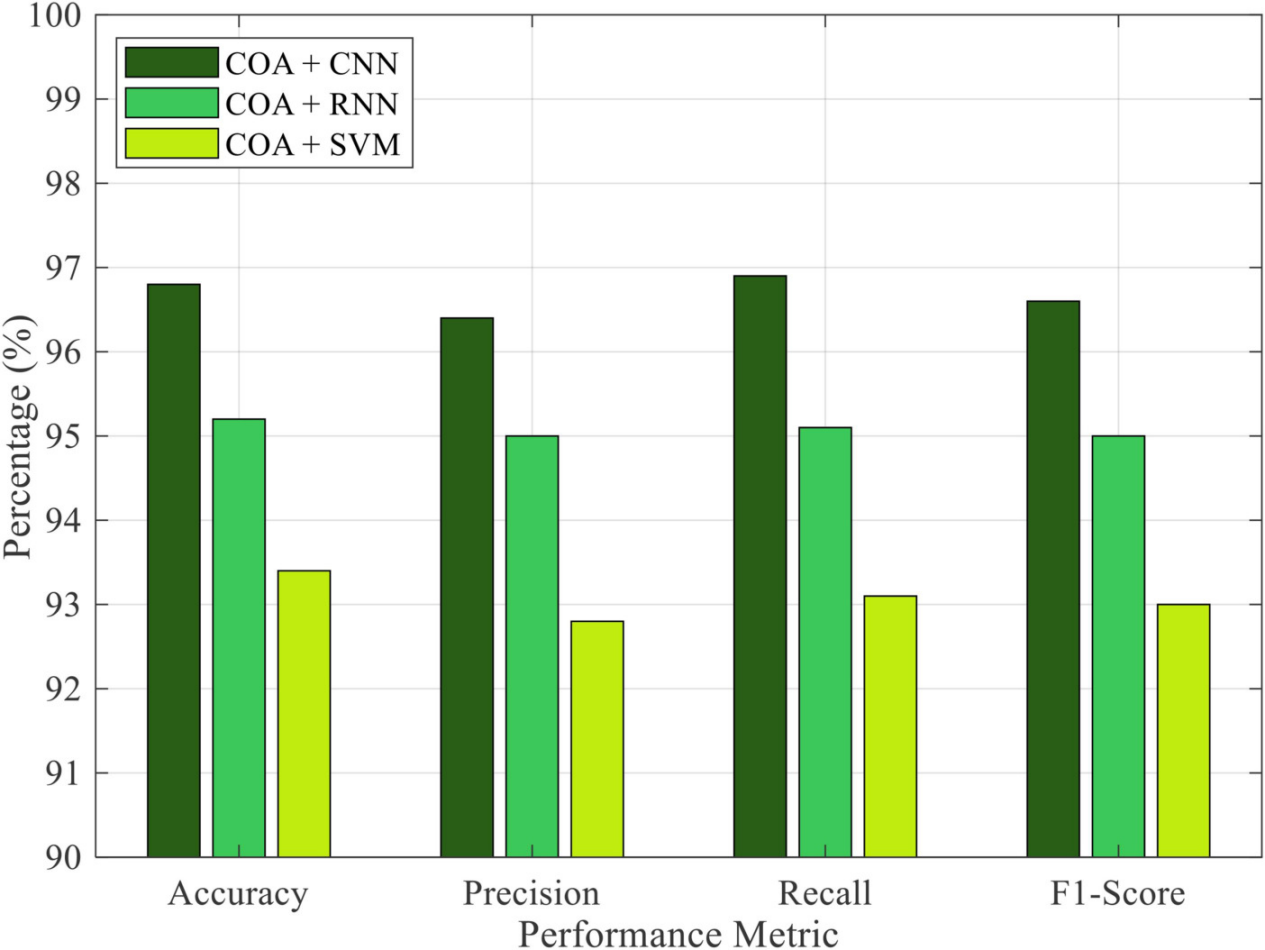
Performance comparison of COA with various classifiers.

The proposed method includes a performance comparison of the proposed COA-based feature selection framework integrated with three different classifiers: CNN, RNN, and SVM, as shown in Figure 1. Among these classifiers, the COA + CNN configuration outperforms the others, achieving the highest accuracy of 96.8%, a precision of 96.4%, a recall of 96.9%, and an F1-score of 96.6%. This finding suggests that the CNN is highly effective at learning spatially localized patterns in EEG signals, making it ideal for neural prosthetic control applications.

The COA + RNN model also demonstrates strong performance, achieving an accuracy of 95.2% by leveraging temporal dependencies in EEG sequences. However, its slightly lower precision and recall values compared to CNN indicate reduced stability in classifying motor imagery intentions. On the other hand, the COA + SVM model, although still competitive with 93.4% accuracy, falls short in all metrics compared to the deep learning approaches. This finding highlights the limitations of traditional classifiers in handling high-dimensional and non-linear EEG features, despite optimal feature selection by COA. Therefore, these results confirm that deep learning classifiers, especially CNNs, when combined with COA-based feature optimization, offer a powerful strategy for accurate and robust EEG signal classification in real-time neural prosthetic systems.

### 6.2 Comparison with existing optimization methods

**Figure 2:**
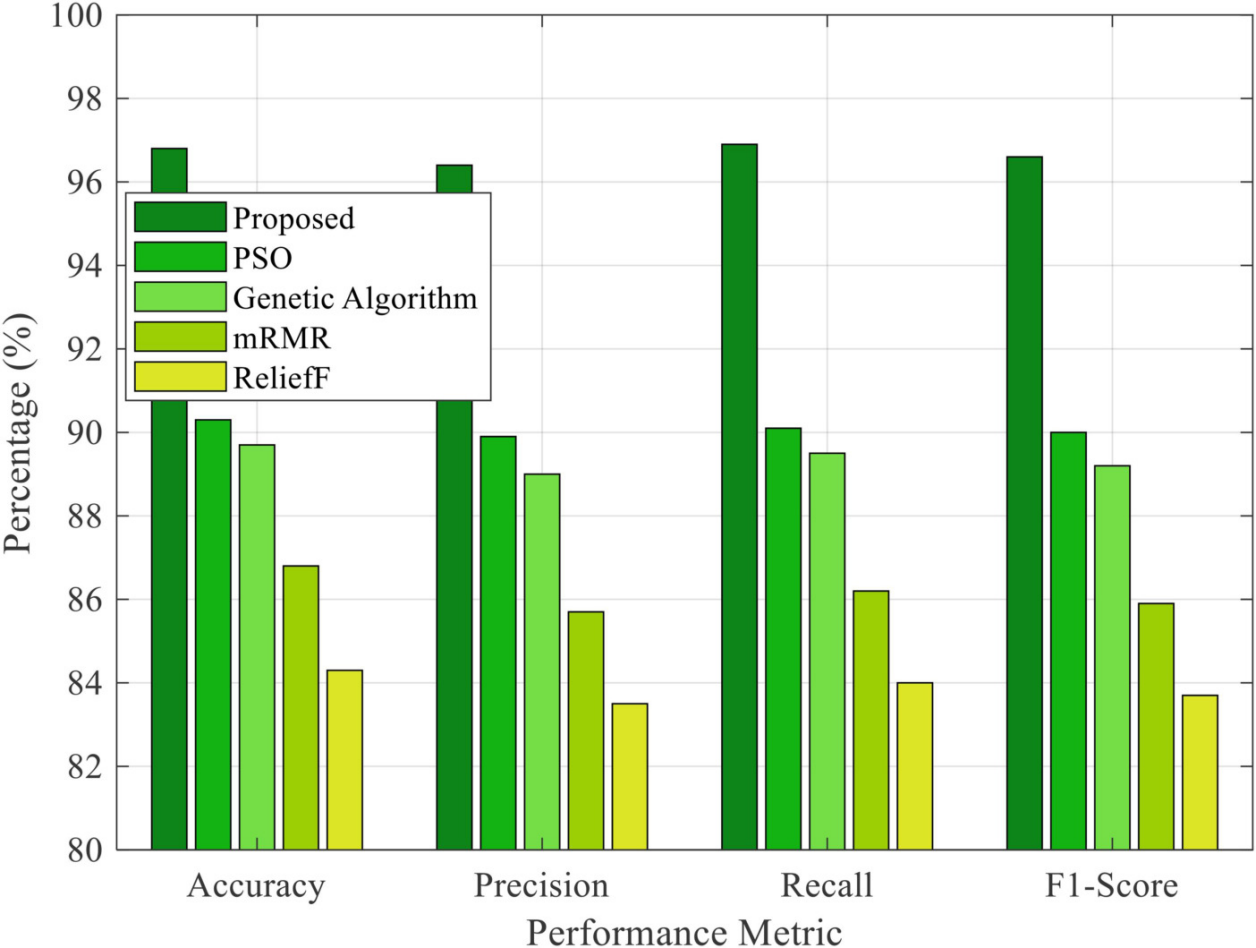
Performance comparison across classification methods.

Figure 2 shows that the comparative analysis of classification performance across the proposed, PSO [12], GA [13], mRMR [6], and ReliefF [7] methods demonstrates the clear superiority of the proposed framework. Achieving an accuracy of 96.8%, a precision of 96.4%, a recall of 96.9%, and an F1-score of 96.6%, the proposed method significantly outperforms all other techniques across all four performance metrics. This finding indicates its exceptional ability to select relevant EEG features and classify motor intentions with high sensitivity and reliability. The PSO and GA, while showing moderate effectiveness with accuracies of 90.3% and 89.7%, respectively, fall short in comparison, suggesting less efficient balance between exploration and exploitation in high-dimensional feature spaces. The classical filter-based methods, like mRMR and ReliefF, yielded comparatively lower performance, with accuracies of 86.8% and 84.3%, respectively, highlighting their limitations in handling the non-linear and noisy nature of EEG signals. Overall, the results confirm that integrating the COA with advanced classifiers provides a robust and efficient framework for accurate EEG signal classification, making it suitable for real-time neural prosthetic control applications.

### 6.3 Analysis of convergence

**Figure 3:**
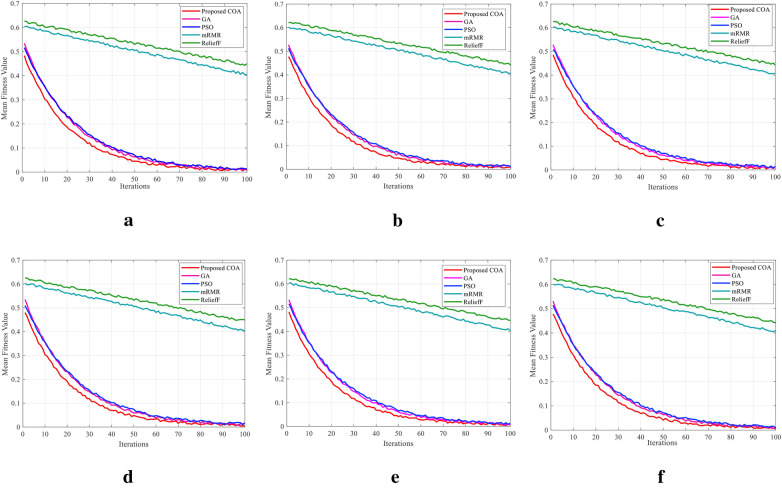
(**a**) PhysioNet motor imagery, (**b**) UCI epileptic seizure, (**c**) EEG eye state, (**d**) BCI Comp II–III, (**e**) Bonn EEG, and (**f**) BCI Comp III–IVa.

The convergence curves shown in Figure 3 across multiple EEG datasets provide valuable insights into the optimization behavior and efficiency of the proposed COA compared with existing methods. Across all datasets, the COA demonstrates a faster and more stable convergence toward the optimal fitness value, typically within the first 30–40 iterations.

This rapid convergence indicates the effectiveness of exploration and exploitation mechanisms, allowing the COA to identify high-quality feature subsets with minimal computational overhead. In contrast, PSO [12] and GAs [13] exhibit slower and more oscillatory convergence patterns, reflecting delayed stabilization and potential premature convergence issues. Traditional feature selection techniques, like mRMR [6], and ReliefF [7], although not iterative optimization methods, are included as baseline references and do not naturally exhibit the iterative convergence trend. However, their fixed performance levels highlight their inability to adaptively improve over iterations. Notably, the COA consistently attains superior fitness values across all datasets, reinforcing its capability to minimize classification error through optimal feature subset selection. The smooth and monotonic nature of COA convergence curves further supports its reliability and robustness, making it particularly well-suited for real-time EEG classification tasks where both computational efficiency and predictive accuracy are essential.

### 6.4 Analysis of the boxplot

**Figure 4:**
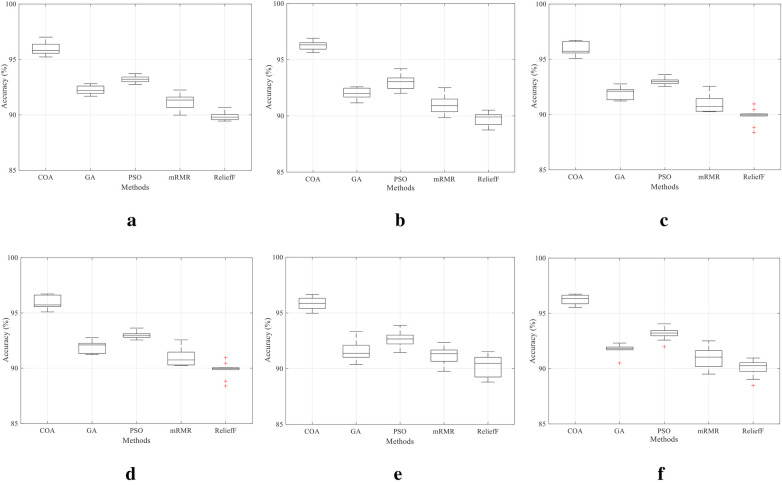
(**a**) PhysioNet motor imagery, (**b**) UCI epileptic seizure, (**c**) EEG eye state, (**d**) BCI Comp II–III, (**e**) Bonn EEG, and (**f**) BCI Comp III–IVa.

The boxplot comparison across multiple EEG datasets illustrates the robustness, consistency, and relative performance of the proposed COA-based feature selection framework relative to conventional methods such as PSO [12], GA [13], mRMR [6], and ReliefF [7], as shown in Figure 4. For each dataset, the boxplot displays the distribution of accuracy scores obtained from multiple experimental runs, highlighting both the central tendency and variability in the performance of each method.

Across all six datasets, the proposed method consistently exhibits higher median accuracy values, narrower inter-quartile ranges, and minimal outliers, indicating both superior classification accuracy and low variance. In contrast, traditional approaches like mRMR and ReliefF show wider spreads and lower medians, suggesting performance instability and reduced adaptability to the diverse EEG data characteristics. Notably, PSO and GAs perform moderately well but still fail to maintain consistency across datasets. These visual insights in Figure 4 reinforce that the COA-based method not only achieves better average performance but also maintains strong stability and generalization across different participants and motor imagery tasks—an essential criterion for real-time neural prosthetic applications.

This investigation demonstrates that deep learning models, owing to their ability to learn complex spatio-temporal patterns, offer significant advantages over traditional classifiers for EEG signal decoding. Personalized calibration and adaptive learning strategies are recommended to mitigate inter-subject variability and ensure consistent control accuracy in practical neural prosthetic applications. In addition, integration with optimization algorithms such as the COA can improve the efficiency of feature selection, reduce computational complexity, and improve classification robustness.

## 7 Conclusion and future scope

In this paper, we proposed a COA-based framework for EEG signal classification tailored to neural prosthetic applications. The integration of COA with deep classifiers yielded high-quality feature selection and significantly improved classification performance. The proposed method achieved an accuracy of 96.8%, a precision of 96.4%, a recall of 96.9%, and an F1-score of 96.6%, outperforming conventional algorithms such as PSO (90.3% accuracy), GA (89.7%), mRMR (86.8%), and ReliefF (84.3%). The convergence analysis confirmed the proposed method has a faster and more stable optimization trajectory, reaching near-optimal solutions, while other methods showed slower or fluctuating patterns. Boxplot comparisons across six benchmark EEG datasets further demonstrated the reliability and robustness of the method, with narrow distributions and higher median accuracy in all cases. These results established the proposed framework as a generalized solution for real-time EEG-based motor intention classification, offering a reliable foundation for advancing intelligent neural prosthetic systems.

Future work may focus on incorporating transfer learning and domain adaptation to improve cross-participant generalization, minimizing the need for personalized training. Expanding the framework to handle multiclass motor imagery tasks and integrating it into low-power embedded platforms would further facilitate its deployment in real-world assistive technologies. Finally, extensive real-time trials with users, particularly individuals with motor impairments, will be essential to validate the practicality, robustness, and long-term reliability of the system.

## Data Availability

The original contributions presented in the study are included in the article/Supplementary Material; further inquiries can be directed to the corresponding author/s.
