# Correction: Advanced EEG signal classification for neural prosthetic devices using metaheuristic and deep learning techniques

**DOI:** 10.3389/fdgth.2026.1795848

**Published:** 2026-03-09

**Authors:** Thippagudisa Kishore Babu, Damodar Reddy Edla, Suresh Dara, Mohan Allam

**Affiliations:** 1 Department of Computer Science and Engineering, National Institute of Technology, Cuncolim, Goa, India; 2 School of Computer Science and Engineering, VIT-AP University, Amaravati, Andhra Pradesh, India

**Keywords:** coati optimization algorithm (COA), deep learning, EEG signal classification, feature selection, motor imagery, neural prosthetic devices

Author Suresh Dara was erroneously assigned as corresponding author. The correct corresponding author is Mohan Allam whose email has been updated.

The original version of this article has been updated.

